# The Modern Treatment of Cancer

**Published:** 1906-12-29

**Authors:** 


### The Hospital

A Journal of

The Medical Sciences and Hospital Administration.

Vol. XLI.—No. 1,059. SATURDAY, DECEMBER 29, 1906.

### The Modern Treatment of Cancer.

The discussion of the various aspects and relationships of the problem of cancer engages public not less than professional attention. Nor is this by any means matter for surprise. The disease is so widespread, that directly or indirectly many, or even most, persons know of individual victims. Its malignant nature and ruthless course compel human interest and sympathy. And the hope and expectation of the discovery of a means of checking its ravages are ever ready to quicken into active and confident emotion. It is true, indeed, of all diseases, but is specially true of the developments of cancer, that the risks and liabilities they carry for the individual, and the sense of tenderness they stir towards their victims, make them public questions in the best sense of that term. So far from this interest being obnoxious to the medical profession the very opposite is the case. Successful progress in the solution of the cancer problem demands both sympathy and financial support, and for these the profession must look mainly to members of the lay public. For these reasons, and also because of the more general consideration that the more the intelligent public understands the motives and methods of medicine the better for all concerned, we can see no objection to frank and well-informed statements on the question of cancer appearing in the lay Press. Granted that such statements are accurate and are presented with due caution, their influence in the education of the public must be wholly in the right direction.

It is impossible to think without a sense of horror and of indignation of the cruel bitterness of disappointed hope that has followed, in the experiences of multitudes of sufferers, the sensational paragraphs produced in the lay Press on the subject of cancer in recent years. Our objection is not to publicity, but to the presentation to sufferers and to their anxious friends of statements which have not the warrant of experience to sustain them. The experimental and tentative stage of any method of treatment should be discussed among those competent to appreciate results and to weigh evidence, and not among an anxious and untrained public.

On a very different plane from newspaper propaganda is Mr. Edmund Owen's Bradshaw lecture. Of great practical value and interest to the surgeon, its authoritative announcements are of importance to the whole of the profession, and even to the general public. Its final verdict on the present position is stated with no uncertain sound. "In the present state of medical and surgical knowledge," says Mr. Owen, "the only way in which the cure of a cancer can be obtained is by its thorough and prompt removal." Further—and here again the public, not less than the profession, may well give heed—when a growth has once been recognised as malignant, time is a factor of the highest importance. Delay means added risk. So also does limitation of the extent of the operation. The proclamation, therefore, is surgery, early surgery, and extensive surgery. Qualify or interfere with this in any degree, and the risks of recurrence, which exist even in the most fortunate circumstances, are immediately multiplied. It is, indeed, a severe and terrible conclusion. Yet who, knowing the facts, can doubt its abundant justification? And, this being so, it is obvious that whilst harsh in form it is kind and gentle in substance. Not only so, but in view of the extent to which successful treatment, as above defined, depends upon early recognition and prompt operation, the statement of the true position of affairs is a humane discharge of a terrible responsibility. Mr. Owen's conclusion does not, of course, rest merely on his own experience, considerable as that is known to be. It has for its support the emphatic endorsement of all experienced surgeons, whether in hospital or private practice. In these circumstances who will envy the responsibility of him who would persuade the public that an alternative to the knife exists, and would thus invite to the delay which experienced surgery pronounces to be full of danger?

From what has just been said it is the plain duty of the medical practitioner to urge immediate operation as soon as the diagnosis of malignant disease has been made, assuming, that is, that the growth is within the compass of surgical interference. This does not imply that there is no field for experimental therapeutics in connection with cancer. There are, indeed, only too many inoperable cases, and in these we quite agree that any reputed remedy not only might be, but ought to be, employed. The profession is fully awake to this claim, and a considerable amount of observation on these lines has been in active progress during recent years. If out of this there should come a real "cure" for cancer, fortunate indeed will be the issue. But in the meantime, there is no reliable help short of operation, and the obvious responsibility of everyone concerned is to affirm this in no hesitating or uncertain terms.

